# Important drug classes associated with potential drug–drug interactions in critically ill patients: highlights for cardiothoracic intensivists

**DOI:** 10.1186/s13613-015-0086-4

**Published:** 2015-11-24

**Authors:** Shadi Baniasadi, Behrooz Farzanegan, Maryam Alehashem

**Affiliations:** Virology Research Center, National Research Institute of Tuberculosis and Lung Diseases (NRITLD), Shahid Beheshti University of Medical Sciences, Tehran, Iran; Tracheal Diseases Research Center, National Research Institute of Tuberculosis and Lung Diseases (NRITLD), Shahid Beheshti University of Medical Sciences, Tehran, Iran

## Abstract

**Background:**

Patients in the intensive care unit (ICU) are more prone to drug–drug interactions (DDIs). The software and charts that indicate all interactions may not be proper for clinical usage. This study aimed to identify the main drug classes associated with clinically significant DDIs in cardiothoracic ICU and categorize DDIs to make cardiothoracic intensivists aware of safe medication usage.

**Methods:**

This prospective study was conducted over 6 months in a cardiothoracic ICU of a university-affiliated teaching hospital. The presence of potential drug–drug interactions (pDDIs) was assessed by a clinical pharmacologist using Lexi-Interact database. Clinically significant pDDIs were defined according to severity and reliability rating. Interacting drug classes, mechanisms, and recommendations were identified for each interaction.

**Results:**

From 1780 administered drugs, 496 lead to major (D) and contraindicated (X) interactions. Nine drug classes were responsible for D and/or X interactions with excellent (E) and/or good (G) reliability. Anti-infective agents (45.87 %) were the main drug classes that caused clinically significant pDDIs followed by central nervous system drugs (14.67 %). Azole antifungals as the most interacting antimicrobial agents precipitated metabolism inhibition of CYP3A substrates.

**Conclusions:**

Clinically significant pDDIs as potential patient safety risks were prevalent in critically ill patients. The findings from current study help to improve knowledge and awareness of clinicians in this area and minimize adverse events due to pDDIs.

## Background

Drug–drug interactions (DDIs) are a growing concern in all clinical settings, particularly intensive care units (ICUs) [1]. ICU-admitted patients are at an increased risk of DDIs due to the complexity of pharmacotherapy, large number of medications, disease severity, and organ failure [2–5]. DDIs are common causes of adverse drug events that may affect patient health; therefore, the identification and prevention of potential drug–drug interactions (pDDIs) could improve patient safety [6]. Since it would be impossible for any physician to remember all pDDIs, improving the knowledge of clinical practitioners in terms of clinically important DDIs could reduce the risk of serious adverse outcomes. Applying DDI software and employing clinical pharmacists to detect and prevent DDIs have improved patient safety in advanced countries [1, 7], while physicians in developing countries still identify DDIs based on their own experiences [8, 9].

Previous studies regarding pDDIs have focused on frequency, type, mechanisms, severity, drug combinations, management and related outcomes in ICU settings [1, 10–14], but important drug classes involved in well-documented and severe pDDIs in cardiothoracic ICU have not yet been reported. Therefore, the current study aimed to highlight the main drug classes associated with clinically important DDIs. The results of this study could increase knowledge among intensivists (especially cardiothoracic intensivists) in the context of DDI prevention and safe medication usage.

## Methods

### Study design and protocol

This cross-sectional study was conducted at Masih Daneshvari Hospital, a university-affiliated hospital for lung diseases, over a 6-month period. The study protocol was approved by the hospital’s ethical review board. Data related to the pharmacotherapy of the subjects were assessed 48 h after admission to an 8-bed surgical cardiothoracic ICU by a clinical pharmacologist using Lexi-Interact database, a complete drug and herbal interaction analysis program. The software identifies interacting drugs, mechanisms, severity, reliability (documentation) rating (E = excellent, G = good, F = fair), potential outcomes, and clinical management. According to severity, the interactions are categorized into five categories: A (unknown), B (minor), C (moderate), D (major), and X (contraindicated) [15]. Drugs involved in pDDIs and subjects’ diagnoses were classified according to AHFS Drug Information [16] and International Classification of Diseases (ICD10), respectively [17]. At the time of admission, any home medication, herbal medication, and nutritional supplements were stopped according to the ICU policy. Hydro-electrolytic components, insulin, water-soluble vitamins, and topical drugs were excluded from further analysis. Physicians were notified of interactions that might lead to alterations in drug therapy.

### Data analysis

Data were analyzed using descriptive statistics from the SPSS software v.22.0 for Windows (SPSS, Chicago, IL). The median, range, and percentage were applied to present the results where appropriate. The interacting drug classes, significance, reliability, and clinical management of the interactions were recorded in a database. Interactions with a severity rating of D and/or X and a reliability rating of E and/or G were considered clinically important pDDIs for more analysis.

## Results

Overall, 195 prescriptions were evaluated over the 6-month study period. Table 1 shows the demographic and clinical characteristics of the subjects. The average length of stay in ICU was 5 days. Total administered and interacting medications (D and X interactions with any documentation rating) were 1780 and 496, respectively (Table 2). D and/or X interactions were identified in 37.94 % (74) of the prescriptions. Of the 248 pDDIs with a severity rating of D and/or X, 157 were unique drug pairs (a specific combination of interacting medication that is counted one time). The drug classes and specific medications involved in D and/or X interactions are shown in Table 3. The drug pairs causing D and/or X interactions with E and/or G reliability are displayed in Table 4.

**Table 1:** Demographic and clinical characteristics of the patients

Characteristics	
Number of patients (*n*)	184
Age [median (range)]	48 (3–85)
Gender (male/female)	110/74
Number of prescriptions (*n*)	195
Number of prescriptions including:	
1–8 drugs (*n*)	110
9–17 drugs (*n*)	71
18–25 drugs (*n*)	14
Diagnoses classification (*n*)	
Certain infectious and parasitic diseases	7
Neoplasms	43
Diseases of the blood and blood-forming organs and certain disorders involving the immune mechanism	1
Diseases of the circulatory system	32
Diseases of the respiratory system	68
Diseases of the digestive system	6
Congenital malformations, deformations and chromosomal abnormalities	6
Symptoms, signs and abnormal clinical and laboratory findings, not elsewhere classified	1
Injury, poisoning and certain other consequences of external causes	4
Factors influencing health status and contact with health services	16

**Table 2:** The number and percentages of total administered and interacting drugs in different drug classes

Drug classes	Total administered drugs % (*n*)	D and/or X interacting drugs % (*n*)	D and/or X interacting precipitant drugs with E and/or G reliability % (*n*)
Anti-infective agents	22.35 % (398)	25.00 % (124)	45.87 % (50)
Central nervous system agents	20.50 % (365)	19.95 % (99)	14.67 % (16)
Cardiovascular drugs	19.21 % (342)	13.10 % (65)	5.50 % (6)
Gastrointestinal drugs	12.80 % (228)	6.65 % (33)	4.58 % (5)
Hormones and synthetic substitutes	6.68 % (119)	11.29 % (56)	2.75 % (3)
Respiratory tract agents	6.40 % (114)	3.22 % (16)	–
Electrolytic, caloric, and water balance	5.33 % (95)	6.65 % (33)	10.09 (11)
Blood formation, coagulation, and thrombosis	2.07 % (37)	3.42 % (17)	3.67 % (4)
Autonomic drugs	2.02 % (36)	3.02 % (15)	9.17 % (10)
Miscellaneous therapeutic agents	2.02 % (36)	7.05 % (35)	3.66 % (4)
Vitamins	0.56 % (10)	0.60 % (3)	–
Total	1780	496	109

**Table 3:** Drug classes, specific medications associated with D and/or X interactions, and the frequency of interactions

Drug classes (frequency of interactions)	Specific medications (frequency of interactions)
Anti-infective agents (124)	Itraconazole (24), Ciprofloxacin (17), Voriconazole (16), Rifampin (14), Clarithromycin (11), Erythromycin (10), Dapsone (7), Co-trimoxazole (5), Levofloxacin (4), Posaconazole (4), Meropenem (3), Ofloxacin (3), Isoniazid (2), Pyrazinamide (1), Lamivudine (1), Valganciclovir (1), Caspofungin (1)
Central nervous system agents (99)	Carbamazepine (20), Phenytoin (16), Fentanyl (11), Midazolam (10), Haloperidol (8), Valproic acid (5), Indomethacin (5), Quetiapine fumarate (4), Citalopram (4), Risperidone (2), Imipramine (2), Clonazepam (2), Methadone (2), Lorazepam (1), Chlorpromazine (1), Celecoxib (1), Diclofenac (1), Fluvoxamine (1), Alprazolam (1), Clomipramine (1), Carbidopa and Levodopa (1)
Cardiovascular drugs (65)	Atorvastatin (19), Metoprolol (10), Losartan (6), Amiodarone (6), Amlodipine (4), Diltiazem (4), Captopril (3), Sildenafil (2), Carvedilol (2), Furosemide (2), Digoxin (2), Propranolol (1), Dabigatran (1), Heparin (1), Enalapril (1), Gemfibrozil (1)
Hormones and synthetic substitutes (56)	Dexamethasone (33), Prednisolone (9), Methylprednisolone (6), Levothyroxine (4), Hydrocortisone (3), Octreotide (1)
Miscellaneous therapeutic agents (35)	Cyclosporine (15), Tacrolimus (10), Mycophenolate (6), Tamsulosin (3), ALLOPURINOL (1)
Electrolytic, caloric, and water balance (33)	Calcium carbonate (16), Magnesium sulfate (5), Sodium polystyrene sulfate (4), Calcium gluconate (3), Sodium phosphate (2), Spironolactone (1), Zinc sulfate (1), Potassium chloride (1)
Gastrointestinal drugs (33)	Pantoprazole (19), Granisetron (8), Ranitidine (3), Metoclopramide (1), Omeprazole (1), Aluminum, Magnesium hydroxide (1)
Blood formation, coagulation, and thrombosis (17)	Warfarin (6), Clopidogrel (4), Ferrous sulfate (4), Acetylsalicylic acid (3)
Respiratory tract agents (16)	Ipratropium (7), Formoterol (3), Fluticasone (3), Salbutamol (2), Theophylline (1)
Autonomic drugs (15)	Atracurium (9), Tizanidine (2), Prazosin (2), Cisatracurium (1), Epinephrine (1)
Vitamins (3)	Calcitriol (3)

**Table 4:** Drug classes, precipitant and object drugs, mechanisms and recommendations for clinically significant pDDIs

Drug classes	Precipitant drugs^a^	Object drugs^a^	Mechanisms	Recommendations
Anti-infective agents	Itraconazole	Tacrolimus	Metabolism (inh)	Monitor tacrolimus concentration
		Diltiazem	Metabolism (inh)	Monitor diltiazem toxic effects
		Digoxin	Absorption	Monitor digoxin concentration
		Atorvastatin	Metabolism (inh)	Reduce dose, monitor toxic effects
		Clarithromycin	Metabolism (inh)^b^	Monitor toxic effect of clarithromycin/itraconazole
		Methylprednisolone	Metabolism (inh)	Reduce dose, monitor corticosteroid toxicity
	Voriconazole	Tacrolimus	Metabolism (inh)	Reduce dose, monitor tacrolimus concentration
		Cyclosporine	Metabolism (inh)	Reduce dose, monitor cyclosporine concentration
		Diltiazem	Metabolism (inh)	Monitor diltiazem toxic effects
		Methylprednisolone	Metabolism (inh)	Reduce dose, monitor corticosteroid toxicity
		Methadone	Metabolism (inh)	Monitor methadone toxic effects
		Midazolam	Metabolism (inh)	Monitor midazolam toxic effects
	Ciprofloxacin	Theophylline	Metabolism (inh)	Reduce dose, monitor theophylline toxic effects
		Erythromycin	Additive	Avoid combination
		Formoterol	Additive	Avoid combination
		Granisetron	Additive	Avoid combination
		Voriconazole	Additive	Avoid combination
	Rifampin	Itraconazole	Metabolism (ind/inh)^b^	Avoid combination/monitor for clinical response to itraconazole
		Clarithromycin	Metabolism (ind/inh)^b^	Monitor for clarithromycin therapeutic effects/rifampin toxic effects
		Cyclosporine	Metabolism (ind)	Consider an alternative, monitor cyclosporine serum concentrations
		Midazolam	Metabolism (ind)	Monitor midazolam therapeutic effects
		Dapsone	Metabolism (ind)	Monitor for dapsone therapeutic effects and methemoglobinemia
	Erythromycin	Amlodipine	Metabolism (inh)	Consider an alternative, monitor toxic effects
		Carbamazepine	Metabolism (inh)	Consider an alternative, monitor toxic effects
		Dexamethasone	Metabolism (inh)	Monitor corticosteroids toxic effects
		Prednisolone	Metabolism (inh)	Monitor corticosteroids toxic effects
	Posaconazole	Midazolam	Metabolism (inh)	Consider an alternative, monitor toxic effects
	Isoniazid	Metoprolol	Metabolism (inh)	Consider an alternative, monitor response
	Valganciclovir	Lamivudine	Additive	Monitor hematologic toxicity
	Clarithromycin	Midazolam	Metabolism (inh)	Consider an alternative, monitor for toxic effects
Central nervous system agents	Carbamazepine	Phenytoin	Metabolism (ind)^b^	Monitor for phenytoin/carbamazepine serum concentrations
		Clonazepam	Metabolism (ind)	Consider an alternative
		Warfarin	Metabolism (ind)	Monitor therapeutic effects
		Citalopram	Metabolism (ind/inh)^b^	Monitor citalopram therapeutic effects/carbamazepine toxic effects
		Fluvoxamine	Metabolism (ind/inh)^b^	Monitor fluvoxamine therapeutic effects/carbamazepine toxic effects
	Imipramine	Metoprolol	Metabolism (inh)	Consider an alternative, monitor response
		Citalopram	Metabolism (inh)^b^	Consider an alternative, monitor toxic effects of imipramine/citalopram
	Quetiapine fumarate	Haloperidol	Additive	Avoid combination
		Carbidopa and levodopa	Antagonistic	Consider an alternative
	Phenytoin	Amlodipine	Metabolism (ind/inh)^b^	Monitor amlodipine therapeutic effects/phenytoin toxicity
	Indomethacin	Acetylsalicylic acid	Additive	Monitor bleeding, avoid regular use of indomethacin
		Cyclosporine	Additive	Consider an alternative, monitor nephrotoxicity
		Furosemide	Antagonistic	Monitor therapeutic effect
	Valproic acid	Lorazpam	Metabolism (inh)	Monitor lorazepam toxic effects
Electrolytic, caloric, and water balance	Sodium polystyrene sulfate	Calcium carbonate	Antagonistic	Separate doses by 2 or more hours, monitor metabolic alkalosis
	Zinc sulfate	Ofloxacin	Absorption	Separate doses by 2 or more hours
	Magnesium sulfate	Ciprofloxacin	Absorption	Separate doses by 2 or more hours
	Calcium carbonate	Isoniazid	Absorption	Separate doses by 2 or more hours
		Levofloxacin	Absorption	Separate doses by 2 or more hours
		Mycophenolate	Absorption	Separate doses by 2 or more hours
Autonomic drugs	Atracurium	Methylprednisolone	Additive	Monitor neuromuscular adverse effects
		Prednisolone	Additive	Monitor neuromuscular adverse effects
		Dexamethasone	Additive	Monitor neuromuscular adverse effects
		Hydrocortisone	Additive	Monitor neuromuscular adverse effects
	Cisatracurium	Prednisolone	Additive	Monitor neuromuscular adverse effects
Cardiovascular drugs	Amiodarone	Digoxin	Excretion	Reduce digoxin dosage, monitor serum concentration
		Metoprolol	Metabolism (inh)	Consider an alternative, monitor response
	Gemfibrozil	Atorvastatin	Metabolism (inh)	Avoid combination
	Metoprolol	Epinephrine	Additive	Monitor pressor effects of epinephrine
Blood formation, coagulation, ……	Acetylsalicylic acid	Warfarin	Additive	Monitor bleeding
	Ferrous sulfate	Ofloxacin	Absorption	Separate doses by 4 or more hours
		Levothyroxine	Absorption	Separate doses by 2 or more hours
Gastrointestinal drugs	Pantoprazole	Itraconazole	Absorption	Administer itraconazole with an acidic beverage, monitor response
	Ranitidine	Itraconazole	Absorption	Administer itraconazole with an acidic beverage, monitor response
Hormones and synthetic substitutes	Dexamethasone	Clarithromycin	Metabolism (ind/inh)^b^	Consider alternative antimicrobial therapy/monitor for dexamethasone toxic effects
		Itraconazole	Metabolism (ind)	Avoid combination, monitor for therapeutic effects
Miscellaneous	Cyclosporine	Atorvastatin	Metabolism (inh)	Avoid combination
		Mycophenolate	Excretion	Monitor mycophenolate dosing and response to therapy

*inh* Inhibition, *ind* induction

^a^The precipitant drug causes the interaction and the object drug is affected by the interaction

^b^Both medications act on each other

## Discussion

The present study evaluated clinically important pDDIs in terms of drug classes, mechanisms, and recommendations in the cardiothoracic ICU. Scientifically, important measures to decrease the risk of DDIs are computerized prescribing, pharmacotherapy monitoring, and pharmacist participation in the multidisciplinary team [18]. Where these services are not available (e.g., due to budget shortages), continued education and expert studies on pDDIs could improve patient safety. Software that identifies a large number of DDIs with any rating of severity and reliability could result in a lack of attention given to the warnings [19]. Categorizing DDIs by drug classes and highlighting more severe and reliable interactions may increase the tendency of clinicians to prevent clinically significant DDIs.

Results of the current study revealed that anti-infective, central nervous system (CNS), and cardiovascular agents were the main drug classes involved in D and X pDDIs. Antimicrobials were the most administered medications in the studied setting and were responsible for a high number of pDDIs. Anti-infective agents are commonly used in the ICU and are associated with clinically significant interactions [20]. Antithrombotic agents and cardiovascular drugs are usually reported as the most common interacting drug groups in ICU settings [1, 10–12]. Different results may be related to the inclusion of moderate and/or not well-documented pDDIs in other studies. These authors’ previous study also showed that antibiotics were the most commonly implicated drug class for adverse drug reactions [21]. Overestimated usage of antibiotics is an important issue in developing countries where the use of antibiotics requires close monitoring to ensure effective treatment and, ultimately, cost reduction [22].

A wide range of different consequences could be predicted according to DDIs mechanisms. The altered gastrointestinal (GI) absorption of antibiotics and the inhibition/induction of drug metabolizer enzymes are the mechanisms most often associated with antimicrobial interactions [20, 23]. The inhibition of metabolism by azole antifungal agents (most interacting drugs in the current study) may lead to increased tacrolimus and cyclosporine concentrations in transplant patients and the increased toxicity of these medications [24]. On the other hand, metabolism induction usually results in decreased clinical efficacy of other drugs instead of adverse effects. Quinolones and macrolides (with torsadogenic potential) may interact synergically with other medications and result in QTc prolongation and torsades de pointes [25]. It was also found that these mechanisms play the main role in anti-infective interactions that could be managed by adjusting drug administration time, and dosage, or using an alternative medication [23, 25].

CNS drugs were 20.50 and 19.95 % of administered medications and interacting drugs, respectively. These medications are frequently used to control seizures, pain, and anxiety and to treat delirium, depression, and psychotic symptoms in ICU settings [26]. A conducted study in a mixed ICU showed that 40 % of the pDDIs were associated to drugs acting on CNS [27]. In the current study, 14.67 % of D and/or X pDDIs with reliability ratings of E and/or G were related to these agents as a precipitant drug. Carbamazepine was the most frequent CNS drug that interacted with CYP3A substrates (such as phenytoin, clonazepam, and warfarin). Monitoring the therapeutic effects of object drugs and considering an alternative medication (if possible) are recommended as appropriate interventions [28]. In addition to metabolism, additive and antagonistic effects were the main mechanisms for CNS drug interactions in this study.

Electrolytic, caloric, and water balance agents, which include metal ions, accounted for 10.09 % of clinically important pDDIs. Metal ions form complexes with other medications, especially antimicrobial agents, and affect drug absorption. A reasonable recommendation is to administer oral medications at least 1–2 h before, and not within 4 h after, administering the metal ions [23].

In autonomic drug classes, the combination of neuromuscular blocking agents and corticosteroids may lead to further risk of prolonged muscle weakness, including neuropathy, myopathy, and/or paralysis. This has been observed most commonly in the ICU setting, particularly in patients requiring high doses of intravenous steroids [29]. It is recommended to monitor for new onset or worsening of adverse neuromuscular effects and to use a neuromuscular blocking drug only when absolutely necessary [30]. Corticosteroids were administered commonly for tracheal stenosis (15.2 %) and COPD (4.3 %) in the studied setting. After that, neoplasms (23.4 %) and transplants (8.7 %) were the most common indications for corticosteroid administrations.

Although “cardiovascular” and “blood formation, coagulation, and thrombosis” agents accounted for 21.28 % of administered medications and 16.52 % of interacting drugs, only 9.17 % of well-documented D and/or X interactions were related to these groups as precipitant drugs. As mentioned above, pDDIs were classified according to the object and precipitant drugs. In 18.35 % of pDDIs, the drugs belonging to these groups were object drugs. Documentation rating was considered to highlight clinically important pDDIs. A high number of interactions caused by these drug classes were rated as fair documentation. Amiodarone (a CYP2D6 inhibitor) should be considered as a precipitant drug with a large number of interactions. Considering an alternative for one of the interacting agents and monitoring responses are usually recommended [31].

Proton pump inhibitors (PPIs) and histamine-2 receptor antagonists (H_2_RAs) are routinely used for critically ill subjects who are at high risk for stress-related mucosal damage (SRMD) [32]. The pH-raising effect of these GI drugs may decrease absorption and the serum concentration of azole antifungal agents (itraconazole, voriconazole, and posaconazole). Use of the azoles oral solutions (instead of capsule) or administration of the azoles with an acidic beverage could minimize the significance of this interaction [33]. Variations in practices and medications between ICU settings may lead to different pDDIs patterns [1]. Proton pump inhibitors are administered in the currently studied setting to patients with a history of GI disorders, major surgeries, and anticoagulant usage. Although there is lack of firm evidence that PPI reduces GI bleeding compared with H2RA or placebo in ICU patients [34], an international survey of 97 units in 11 countries showed that PPIs were the most common stress ulcer prophylaxis agent, used in 66 % of ICUs (64/97) [35]. Using oral PPIs instead of intravenous injection could be a useful strategy to prevent pDDIs.

In hormone and synthetic substitutes, an important interaction occurred between dexamethasone and CYP3A substrates (clarithromycin and itraconazole). Dexamethasone as a strong CYP3A inducer may decrease the serum concentration and therapeutic effectiveness of these medications [36]. Alternative antimicrobial therapy (when possible) and monitoring patients closely for evidence of diminished clinical response are important preventive measures.

Immunosuppressive drugs classified as miscellaneous therapeutic agents can lead to life-threatening pDDIs in transplant subjects. Cyclosporine has been shown to reduce the AUC of mycophenolic acid (MPA) by inhibiting its glucuronide conjugate mycophenolic acid glucuronide conjugate (MPAG) excretion into the bile. Changes in mycophenolate dosage may be required in cases of concurrent cyclosporine starting, stopping, or dose changing [37]. Studies have shown that the concurrent use of cyclosporine with atorvastatin can increase systemic exposure to the statins and related toxicities such as myopathy and rhabdomyolysis. A statin that is less sensitive to this interaction (e.g., pravastatin or fluvastatin) or an alternative type of LDL-lowering medication should be considered [37, 38].

Several limitations could be considered for the current study. Age is an important factor for DDIs prevalence, and a positive relationship has been found between age and the number of DDIs [9]. The median age of patients in this study (48 years) was lower than that of western countries. Differences between the ICUs in developing and advanced countries could explain this discrepancy. For an appropriate interpretation and application of the ICU data, the diversity among countries including funding, laws, cultural values, and disease prevalence should be considered [39, 40]. In the current study, tracheal stenosis and empyema were the most common respiratory system diseases that lead to ICU admissions. Therefore, several reasons (prevalence of diseases, culture, laws, and life expectancy) may play a role in the low median age of patients in the current study.

The second limitation is using one DDI database that could limit the number of pDDIs. Lexi-Interact is a commonly used resource providing detailed DDI information and was available in the studied hospital. This database with a documentation rating that is useful in recognizing well-documented interactions would limit clinical repercussions. Using different software at the same time, however, could lead to more accurate results [41]. The setting of the current study, the cardiothoracic ICU of a pulmonary referral hospital, is another limitation. A list of drugs approved by hospital formulary committee is specific for an ICU setting. However, commonly used drugs in ICUs are usually considered in critical care drug handbooks and manuals [42, 43]. Therefore, reported interactions related to common drugs could be generalized to other ICUs. The interactions caused by specific medications prescribed in a specialized ICU could also be identified and prevented by similar studies.

## Conclusions

Due to the high prevalence of pDDIs in the ICU setting, the authors recommend more specialized studies on clinically significant pDDIs and continued education based on the results. Highlighting important drug classes and specific medications responsible for the interactions in different settings could increase the knowledge and awareness of clinicians to improve patient safety.

## Abbreviations

ICU: intensive care unit
DDIs: drug–drug interactions
pDDIs: potential drug–drug interactions
CNS: central nervous system
PPIs: proton pump inhibitors
H_2_RAs: histamine-2 receptor antagonists
SRMD: stress-related mucosal damage
GI: gastrointestinal
MPA: mycophenolic acid
MPAG: mycophenolic acid glucuronide conjugate
